# Characterization of a cold-active esterase from *Serratia* sp. and improvement of thermostability by directed evolution

**DOI:** 10.1186/s12896-016-0235-3

**Published:** 2016-01-22

**Authors:** Huang Jiang, Shaowei Zhang, Haofeng Gao, Nan Hu

**Affiliations:** College of Biotechnology and Pharmaceutical Engineering, Nanjing Tech University, Nanjing, 211800 P. R. China; State Key Laboratory of Agricultural Microbiology, College of Life Science and Technology, Huazhong Agricultural University, Wuhan, 430070 P. R. China

**Keywords:** Esterase, Cold-active, Salt-tolerant, Serratia sp, Thermo-stability, Error-prone PCR

## Abstract

**Background:**

In recent years, cold-active esterases have received increased attention due to their attractive properties for some industrial applications such as high catalytic activity at low temperatures.

**Results:**

An esterase-encoding gene (*estS*, 909 bp) from *Serratia* sp. was identified, cloned and expressed in *Escherichia coli* DE3 (BL21). The *estS* encoded a protein (EstS) of 302 amino acids with a predicted molecular weight of 32.5 kDa. It showed the highest activity at 10 °C and pH 8.5. EstS was cold active and retained ~92 % of its original activity at 0 °C. Thermal inactivation analysis showed that the T_1/2_ value of EstS was 50 min at 50 °C (residual activity 41.23 %) after 1 h incubation. EstS is also quite stable in high salt conditions and displayed better catalytic activity in the presence of 4 M NaCl. To improve the thermo-stability of EstS, variants of *estS* gene were created by error-prone PCR. A mutant 1-D5 (A43V, R116W, D147N) that showed higher thermo-stability than its wild type predecessor was selected. 1-D5 showed enhanced T_1/2_ of 70 min at 50 °C and retained 63.29 % of activity after incubation at 50 °C for 60 min, which were about 22 % higher than the wild type (WT). CD spectrum showed that the secondary structure of WT and 1-D5 are more or less similar, but an increase in β-sheets was recorded, which enhanced the thermostability of mutant protein.

**Conclusion:**

EstS was a novel cold-active and salt-tolerant esterase and half-life of mutant 1-D5 was enhanced by 1.4 times compared with WT. The features of EstS are interesting and can be exploited for commercial applications. The results have also provided useful information about the structure and function of Est protein.

## Background

Esterases (EC 3.1.1.3, carboxyl ester hydrolases), lipases (EC 3.1.1.1, triacylglycerol hydrolases) and phospholipase, commonly referred to as lipolytic enzymes, principally catalyze the hydrolysis and synthesis of acyl glycerides and other fatty acid esters [1]. Lipolytic enzymes produced from psychrophilic microorganisms in cold environment could be active and stable at low temperatures as compared to mesophilic enzymes. Esterases have gained immense importance in pharmaceutical, polymer, food, flavor, oleochemical, biofuel and detergent industries [2]. The high catalytic activity of these lipolytic enzymes at low temperatures make these more useful for commercial applications [3]. Recently, many cold-active lipolytic enzymes from psychrophiles and psychrotrophs have been discovered and characterized [4–6]. In esterses, the catalytic triad is commonly formed by Ser, His and Asp residues followed the order Ser-Asp-His and the nucleophilic serine residue is usually embedded in a conserved pentapeptide motif (G-X-S-X-G) [7]. In the past decade, a quick progress achieved in the production of recombinant esterases by directed evolution, mutagenesis, structural analysis, protein engineering [8]. Therefore, attempts have been made to enhance the thermo-stability of lipolytic enzymes by directed evolution [9, 10]. The directed evolution is generally used to generate desired variants and investigate the relationship of structure-function without any detailed structure information [11, 12]. Directed evolution creates molecular diversity by various methods such as error-prone PCR, site-specific saturation mutagenesis and DNA shuffling [13–15]. Recently, an esterase of pig liver origin mutated at F407I was used to resolve the racemic mixture of clopidogrel [16]. Our previous studies also confirmed that a cold active esterase (Est11), produced from *Psychrobacter pacificensis*, was salt tolerant, highly active at low temperatures, and its biochemical characteristics made it important for commercial applications [17]. *Serratia* is a genus of Gram-negative, rod-shaped bacteria and is also a well-known source for chitinase production [18]. Only few reports of esterase from *Serratia* sp*.* are available in literature*.*

In this study, a gene encoding a cold-active esterase, termed EstS, was cloned from the marine bacterium *Serratia* sp. and expressed in *E. coli*. The recombinant enzyme was purified to homogeneity and characterized. Furthermore, the effect of altered amino acid on the thermo-stability of esterase was studied by 3D structural model of esterase and circular dichroism (CD) analysis.

## Results

### Gene cloning and sequence analysis

**Fig. 1:**
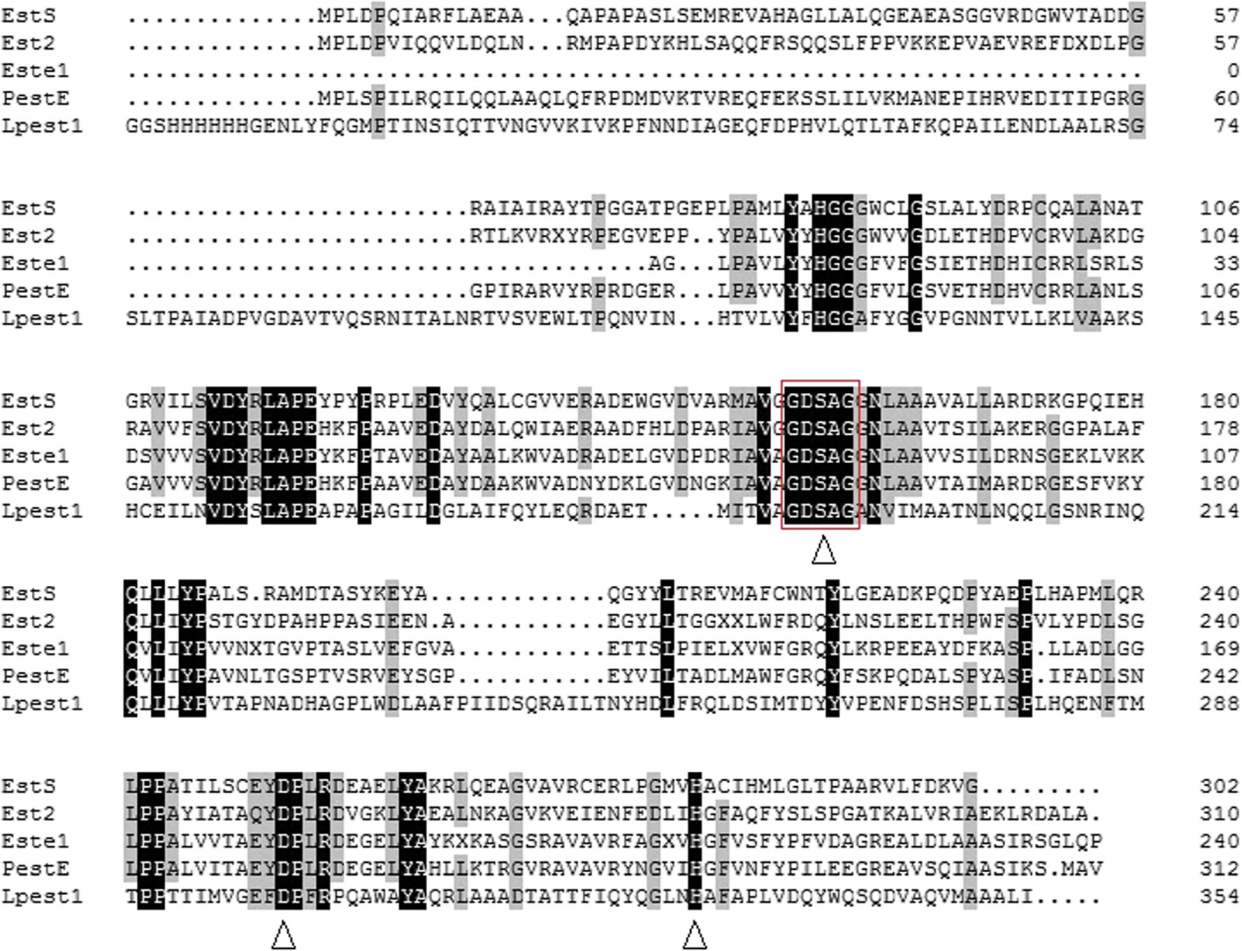
Multiple alignments of EstS and other four esterases. The four esterases are Est2 [PDB: 1EVQ_A] from *Alicyclobacillus Acidocaldarius*, PestE [PDB: 2YH2_A] from *Pyrobaculum calidifontis*, Este1 [PDB: 2C7B_A] from a metagenomic library and Lpest1 [PDB: 4C88_A] from *Lactobacillus Plantarum*. The identical and conserved residues are shaded. The conserved G–X–S–X–G motif and the catalytic triad (Ser, Asp, and His) were indicated by red box and black triangle, respectively

The esterase gene, *estS*, was successfully cloned from *Serratia* sp. genomic DNA. The gene was 909 bp long and encoded a protein (EstS) of 302 amino acids with a theoretical molecular weight of 32.5 kDa. The SignalP 4.1 Server predicted no signal peptide for EstS. BLASTP revealed that the translated protein sequences of EstS showed a high sequence identity to esterase [GenBank: WP_020827011.1] from *Serratia liquefaciens* ATCC 27592 (identity, 98 %), esterase [GenBank: WP_044551387.1] from *Serratia liquefaciens* FK01 (92 %), and esterase [GenBank: WP_037415500.1] from *Serratia grimesii* (82 %). A multiple sequence alignment of EstS was performed with thermophilic carboxylesterase Est2 [PDB: 1EVQ_A] from *Alicyclobacillus acidocaldarius* (identity: 41 %), esterase PestE [PDB: 2YH2_A] from *Pyrobaculum calidifontis* (41 %), carboxylesterase Este1 [PDB: 2C7B_A] from a metagenomic library (40 %), esterase Lpest1 [PDB: 4C88_A] from *Lactobacillus plantarum* (29 %), and all of them were retrieved using BLASTP in the NCBI and PDB database (Fig. 1). The classical catalytic triad consisting of Ser 157, Asp 252 and His 282 was identified and the active site Ser 157 residue was located within the conserved pentapeptide motif (Gly-X-Ser-X-Gly).

### Screening of random mutant library

Screening of random mutant library was based on retention of esterase activity after incubation at high temperature. A clone, designated as 1-D5, which displayed higher thermo-stability than WT enzyme, was selected from over 8000 clones. Sequencing of gene revealed that 1-D5 showed three alterations in amino acid residues (A43V, R116W, D147N).

### Expression and purification

**Fig. 2:**
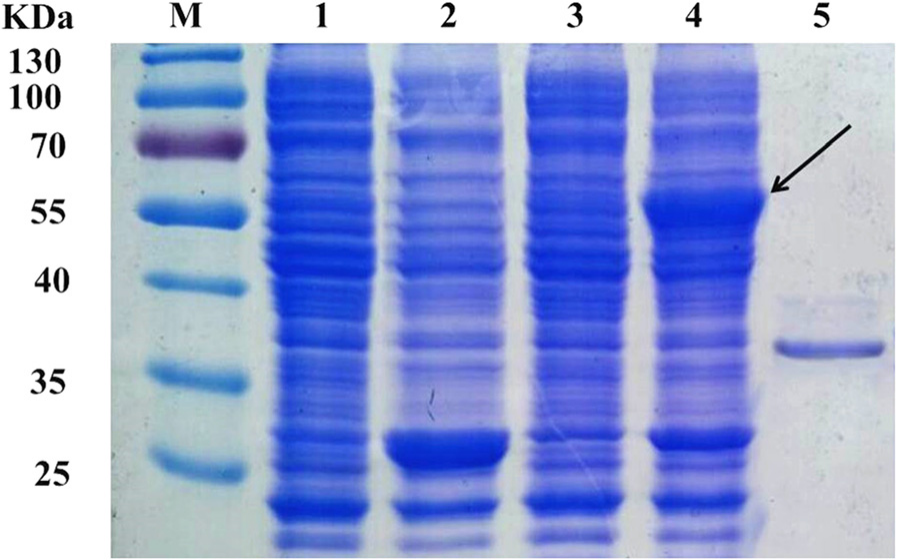
SDS-PAGE analysis of purified EstS protein. M: Protein molecular weight marker; 1: Uninduced cell lysate of *E. coli* BL21 (DE3) harboring pGEX-6P-1; 2: IPTG-induced of cell lysate of *E. coli* BL21 (DE3) harboring pGEX-6P-1; 3: Uninduced cell lysate of *E. coli* BL21 (DE3) harboring pGEX-6P-estS; 4: IPTG-induced of cell lysate of *E. coli* BL21 (DE3) harboring pGEX-6P-estS; 5: Purified EstS. The protein GST-EstS is indicated by arrow

The protein WT and mutant fused with GST tag (58 kDa) were efficiently induced and overexpressed as a soluble, catalytically active form in host strain at 15 °C. The purified WT and mutant (~32 kDa) were detected by SDS-PAGE as a single band, which was consistent with the value predicted from the deduced amino-acid sequence (Fig. 2).

### Substrate specificities

**Fig. 3:**
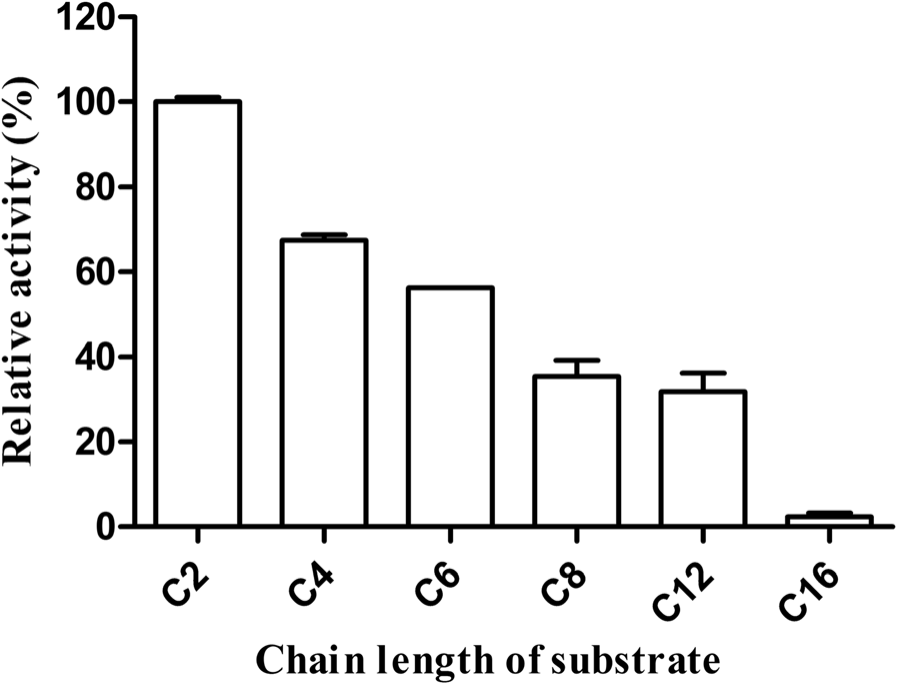
Substrate specificity of the purified EstS. The esterase activity of EstS was tested with various chain lengths of *p*-NP esters (C2, C4, C6, C8, C12 and C16) in 50 mM Tris – HCl, pH 8.5, at 30 °C. The activity against *p*-NP acetate (C2) was taken as 100 %. All measurements were performed in triplicate

The substrate specificity of WT was determined against various aliphatic acyl-chain *p*-NP esters (C2-C16). EstS showed the maximum hydrolytic activity towards *p*-NP acetate (C2), but no activity toward *p*-NP palmitate (Fig. 3). The results indicated that purified protein was an esterase rather than a lipase due to its preference for short acyl-chain *p*-NP esters.

### Biochemical characterization of WT and mutant

**Fig. 4:**
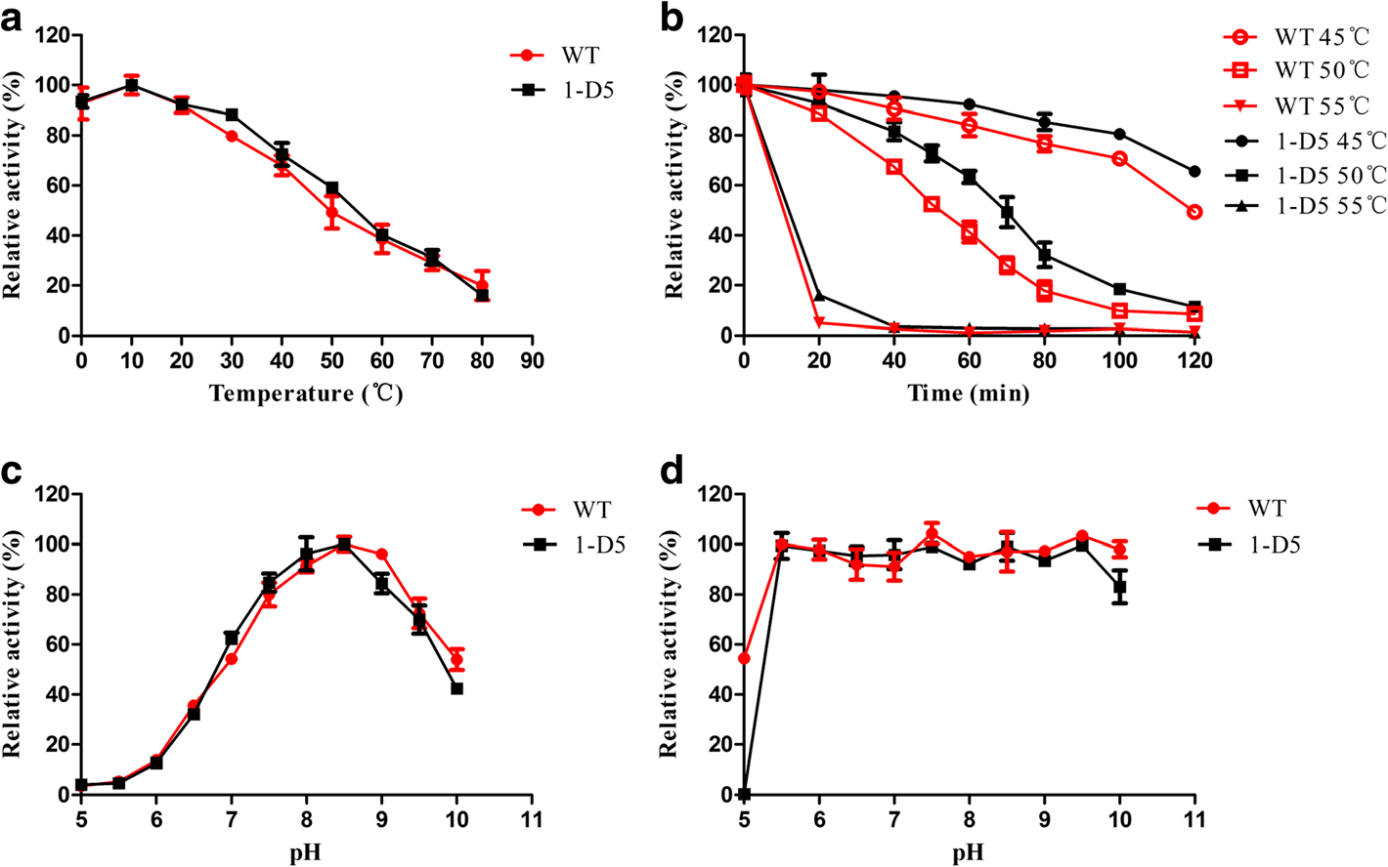
Effect of temperature and pH on enzyme activity and stability of WT and mutant. **a** The effect of temperature on enzyme activity. The temperature-activity profile was measured at a temperature range of 0 to 80 °C in 50 mM Tris–HCl buffer (pH 8.5). Activity value obtained at 10 °C was defined as 100 %. **b** Temperature stability. The WT and mutant enzyme was incubated at 45 (○ WT; ● 1-D5), 50 (□ WT; ■ 1-D5) and 55 °C (▼ WT; ▲ 1-D5) for various time intervals and the residual activity was measured. The specific activity without incubation was taken as 100 %. **c** The effect of pH on enzyme activity. The pH-activity profile was determined in phosphate–citrate buffer (pH 5.0–7.0) and 50 mM Tris–HCl buffer (pH 7.0–10.0) at 10 °C. The activity at pH 8.5 was defined as 100 %. **d** pH stability. The activity was determined by pre-incubating enzyme solutions in different pHs buffers at 4 °C for 24 h and the residual activity was measured under standard condition. The residual activity after treatment with pH 7.0 buffer was shown as 100 %

The optimum activity of WT and mutant was measured over a temperature range of 0–80 °C and a pH range of 5–10 WT showed the maximum activity around 10 °C and retained nearly 92 % activity at 0 °C. The properties of higher hydrolytic activity at a low temperature indicated that EstS was a cold-active enzyme. However, no change in optimum temperature of mutant esterase was observed when compared with WT (Fig. 4a). Thermo-stability analysis showed that T_1/2_ of WT esterase was about 50 min at 50 °C with 41.23 % of its original activity after 1 h incubation. Also, complete loss of WT activity was reported after 20 min incubation at 55 °C (Fig. 4b). In contrast, the mutant enzyme showed enhanced T_1/2_ of 70 min at 50 °C and retained 63.3 % of its initial activity after incubation at 50 °C for 60 min, which were about 22 % higher than WT. The pH activity profile of WT and mutant was examined over the pH range of 5–10 under optimized assay conditions (Fig. 4c). The optimal pH of WT and mutant esterase was found to be 8.5. WT was stable over a wide pH range of 5.5–9.5, but almost inactive at pH 5 when compared with 1-D5 (Fig. 4d).

The effects of various additives on the EstS activity were examined. EstS was slightly activated by 1 mM Mg^2+^ (remaining activity, 121 %), 1 and 5 mM Mn^2+^ (120 %, 114 % respectively), whereas it was fairly inhibited by 1, 5 mM Zn^2+^ (83 %, 71 %) and Cu^2+^ (87 %, 52 %) and 5 mM PMSF (89 %). The mutant 1-D5 showed a similar behavior in the presence of metal ions and relative activity was slightly increased by Mn^2+^ and strongly decreased by the presence of Zn^2+^ and PMSF. However, the activity of EstS was not affected by the presence of EDTA, Ca^2+^, Ba^2+^ and Sr^2+^ (Table 1). The activity of EstS was also strongly reduced by higher concentrations of *iso*propanol, methanol and ethylene glycol (20, 30 %) and was almost completely inhibited by N-butyl alcohol and acetonitrile. Our data also showed that ethanol (10–30 %) increased the activity of the enzyme WT by more than 10 % (Table 2). However, the mutant 1-D5 displayed highest activity in the presence of ethylene glycol (20 % v/v) and a slight decrease in the relative activity was observed in the presence of DMSO and short chain alcohols. Non-ionic detergents such as Triton X-100, Tween 20, Tween 80 and CHAPS showed no significant effect on the enzyme activity of EstS WT and mutant while the anionic detergent SDS almost inactivated it (Table 3).

**Table 1:** Effects of various reagents on the activity of EstS and mutant

Reagents	Relative activity (%)			
	EstS		1-D5	
	1 mM	5 mM	1 mM	5 mM
Control	9.9 ± 0.2 U/ml	8.6 ± 0.5 U/ml	8.1 ± 3.2 U/ml	8.1 ± 3.2 U/ml
Mg^2+^	121.8 ± 1.3	106.5 ± 5.9	95.0 ± 4.4	98.3 ± 1.4
Sr^2+^	102.0 ± 1.7	105.3 ± 2.3	94.2 ± 4.4	101.1 ± 1.1
Ba^2+^	109.7 ± 5.7	107.6 ± 7.4	97.2 ± 3.1	103.2 ± 5.3
Zn^2+^	83.1 ± 3.6	71.6 ± 4.1	39.9 ± 6.3	34.2 ± 4.6
Mn^2+^	120.7 ± 4.0	114.9 ± 4.4	100.1 ± 7.3	106.2 ± 0.8
Cu^2+^	87.6 ± 4.2	52.1 ± 4.0	97.0 ± 7.0	73.9 ± 3.6
Ca^2+^	101.1 ± 1.3	106.2 ± 5.4	98.6 ± 3.5	101.4 ± 3.4
EDTA	96.3 ± 3.6	96.7 ± 1.0	99.1 ± 4.0	97.9 ± 2.6
PMSF	96.4 ± 3.5	89.8 ± 5.0	96.6 ± 4.7	54.8 ± 3.6

Data are given as mean values ± SD. Activity was measured after adding 1 or 5 mM of various reagents into the reaction mixture. The activity without any additives was defined as 100 %

**Table 2:** Effect of organic solvents on the activity of Est WT and 1-D5

Organic solvent	Relative activity (%)					
	EstS			1-D5		
	10 % (v/v)	20 % (v/v)	30 % (v/v)	10 % (v/v)	20 % (v/v)	30 % (v/v)
Control	12.2 ± 0.5 U/ml	10.9 ± 0.2 U/ml	11.2 ± 0.3U/ml	8.2 ± 1U/ml	8.2 ± 1U/ml	8.2 ± 1U/ml
Isopropanol	100.6 ± 4.1	12.0 ± 2.3	6.1 ± 5.7	66.8 ± 1.7	29.1 ± 6.7	14.6 ± 1.6
Acetone	99.6 ± 1.2	103.9 ± 4.0	108.6 ± 2.4	46.2 ± 6.1	19.2 ± 2.1	11.6 ± 0.2
Methanol	112.6 ± 4.6	46.5 ± 4.9	3.9 ± 0.3	79.6 ± 5.2	57.9 ± 4.5	33.0 ± 4.0
DMSO	102.5 ± 5.8	119.8 ± 3.7	108.9 ± 0.3	90.2 ± 5.4	76.9 ± 7.0	40.5 ± 4.5
Ethanol	121.4 ± 3.5	113.7 ± 1.9	110.5 ± 2.5	77 ± 9.7	44.6 ± 3.9	14.3 ± 2.2
N-butyl alcohol	ND	ND	ND	ND	ND	ND
Ethylene glycol	87.8 ± 2.2	83.7 ± 4.3	36.4 ± 5.6	90.9 ± 5.5	93.9 ± 5.9	90 ± 5.2
Acetonitrile	ND	ND	ND	ND	ND	ND

Data are given as mean values ± SD. The activity without any additives was defined as 100 %. ND means not detected

**Table 3:** Effect of detergents on the activity of WT and 1D-5 esterase

Detergents	Relative activity (%)					
	EstS			1-D5		
	0.5 %	1 %	5 %	0.5 %	1 %	5 %
Control	8.8 ± 0.1 U/ml	11.0 ± 0.2 U/ml	10.9 ± 0.3 U/ml	8.4 ± 0.8U/ml	8.4 ± 0.8U/ml	8.4 ± 0.8U/ml
TritonX-100	91.4 ± 1.0	96.3 ± 2.5	90.8 ± 4.5	84.3 ± 5.7	72.0 ± 6.0	39.1 ± 0.6
Tween-20	99.3 ± 1.7	97.7 ± 1.7	96.3 ± 3.8	86.5 ± 5.3	55.4 ± 3.2	45.8 ± 1.3
Tween-80	96.3 ± 2.2	98.4 ± 0.2	96.1 ± 5.5	90.9 ± 4.4	88.8 ± 3.4	83.4 ± 3.6
CHAPS	110.1 ± 2.9	103.8 ± 5.0	3.1 ± 6.4	76.2 ± 5.8	46.6 ± 5.2	26.9 ± 1.3
SDS	ND	ND	ND	ND	ND	ND

Data are given as mean values ± SD. The activity without any additives was defined as 100 %. ND means not detected

**Fig. 5:**
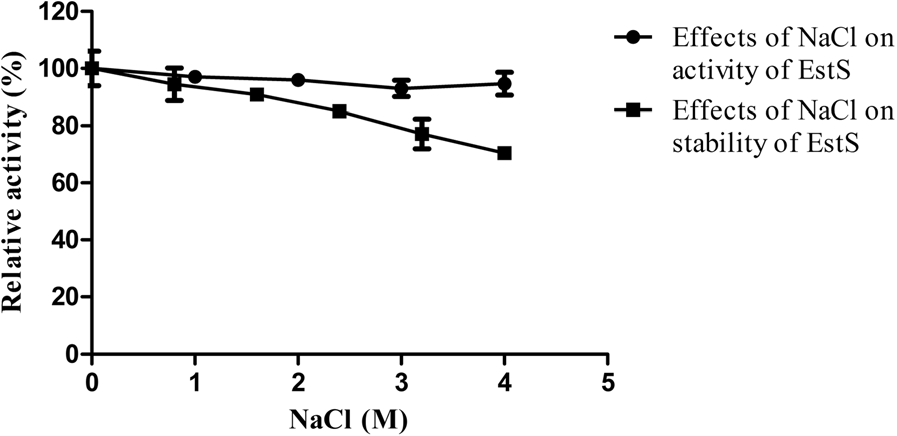
Effects of NaCl on activity and stability of EstS. The enzyme activity (●) was assayed in 50 mM Tris–HCl buffer (pH 8.5) containing 0–4 M NaCl and the residual activity (■) of EstS was measured after incubating with 0–4 M NaCl (pH 8.5) at 4 °C for 24 h

The effect of NaCl on the EstS enzyme activity and stability was further investigated. EstS was not significantly affected and remained robust even at a concentration of 4 M NaCl (Fig. 5). EstS was NaCl- tolerant and retained more than 80 % of its original activity after 24 h incubation at 4 °C in the solution of high salinity (2.4 M NaCl) as depicted in Fig. 5.

### Kinetic measurements

The kinetic parameters of WT and mutant toward *p*-NP acetate were investigated (Table 4). The mutant 1-D5 displayed an 18 % increase in *K*_*m*_ and an 8 % increase in *K*_*cat*_, leading to approximately a 10 % decline in catalytic efficiency *K*_*cat*_*/ K*_*m*_ (Table 4).

**Table 4:** The kinetic parameters of the wild-type EstS and its mutant

Enzyme	*K*_m_ (μM)	*K*_cat_ (s^-1^)	*K*_cat_/*K*_m_ (s^-1^ · μM^-1^)
WT	74.02 ± 1.9	2.339 × 10^3^ ± 4.6	31.60 ± 3.1
1-D5	87.59 ± 2.4	2.501 × 10^3^ ± 6.5	28.55 ± 3.7

Data are given as mean values ± SD. All the assays were performed at the optimum pH and temperature

### Homology modeling

**Fig. 6:**
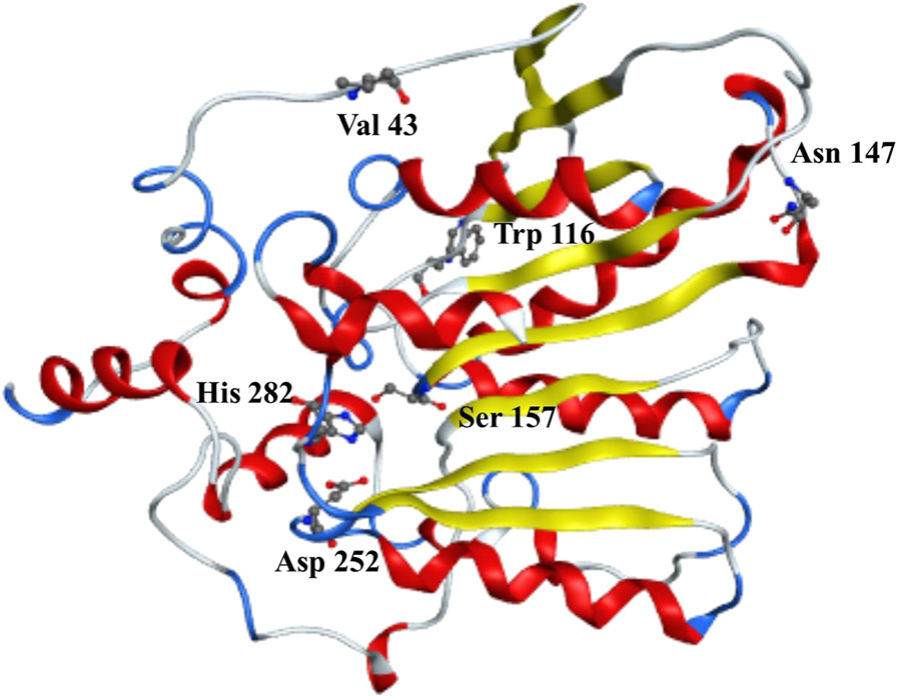
Three-dimensional model of 1-D5. The catalytic sites and substitution sites were displayed with stick-ball model

**Fig. 7:**
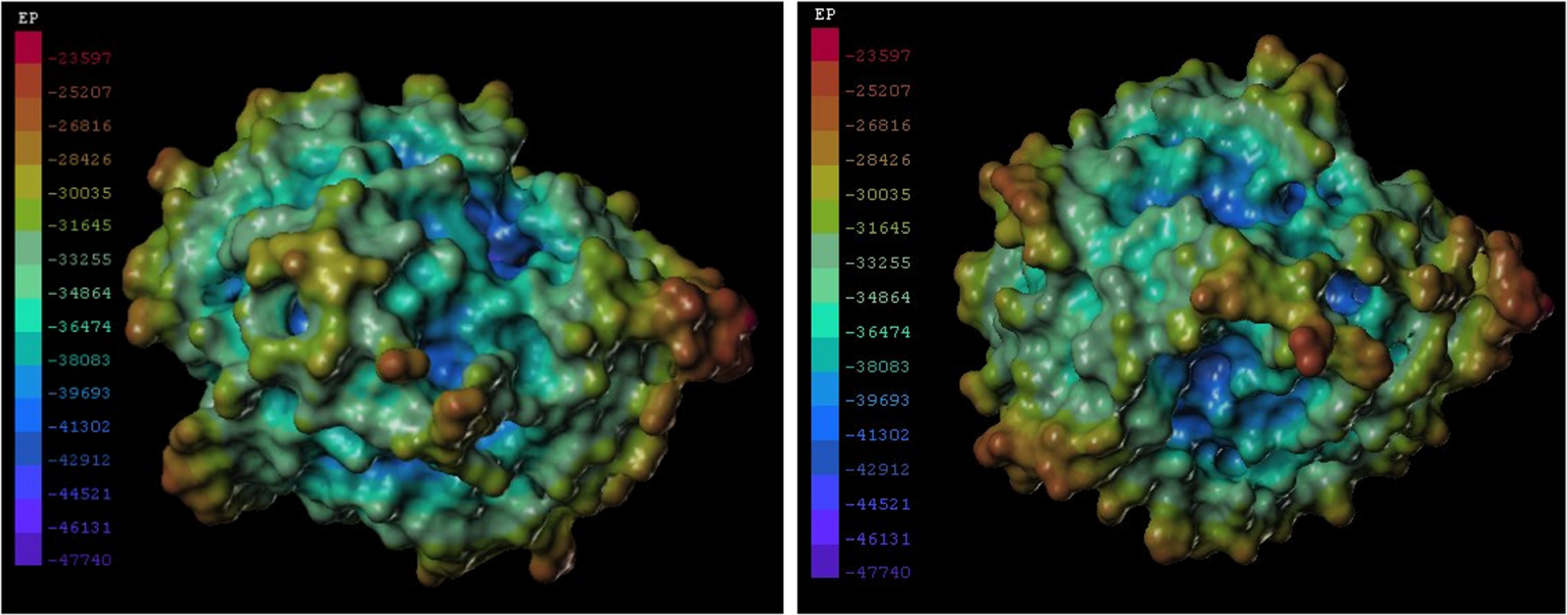
The surface electrostatic potential of EstS. The most negative and most positive electrostatic potentials are indicated by purple and red, respectively. The right image is the 180° rotated view of the left one

The homology models were constructed using swiss-model server with the crystal structure of thermophilic carboxylesterase Est2 [PDB: 1EVQ_A] from *Alicyclobacillus acidocaldarius* as the template (41 % identity to EstS). Both the predicted models of the WT and mutant enzyme exhibit the typical α/β hydrolase fold, which was characteristic of lipolytic enzymes (Fig. 6). The electrostatic potential of EstS was calculated and described (Fig. 7). The distribution of charges revealed that EstS had high negative charges on the surface.

### Circular dichroism and secondary structure prediction

**Fig. 8:**
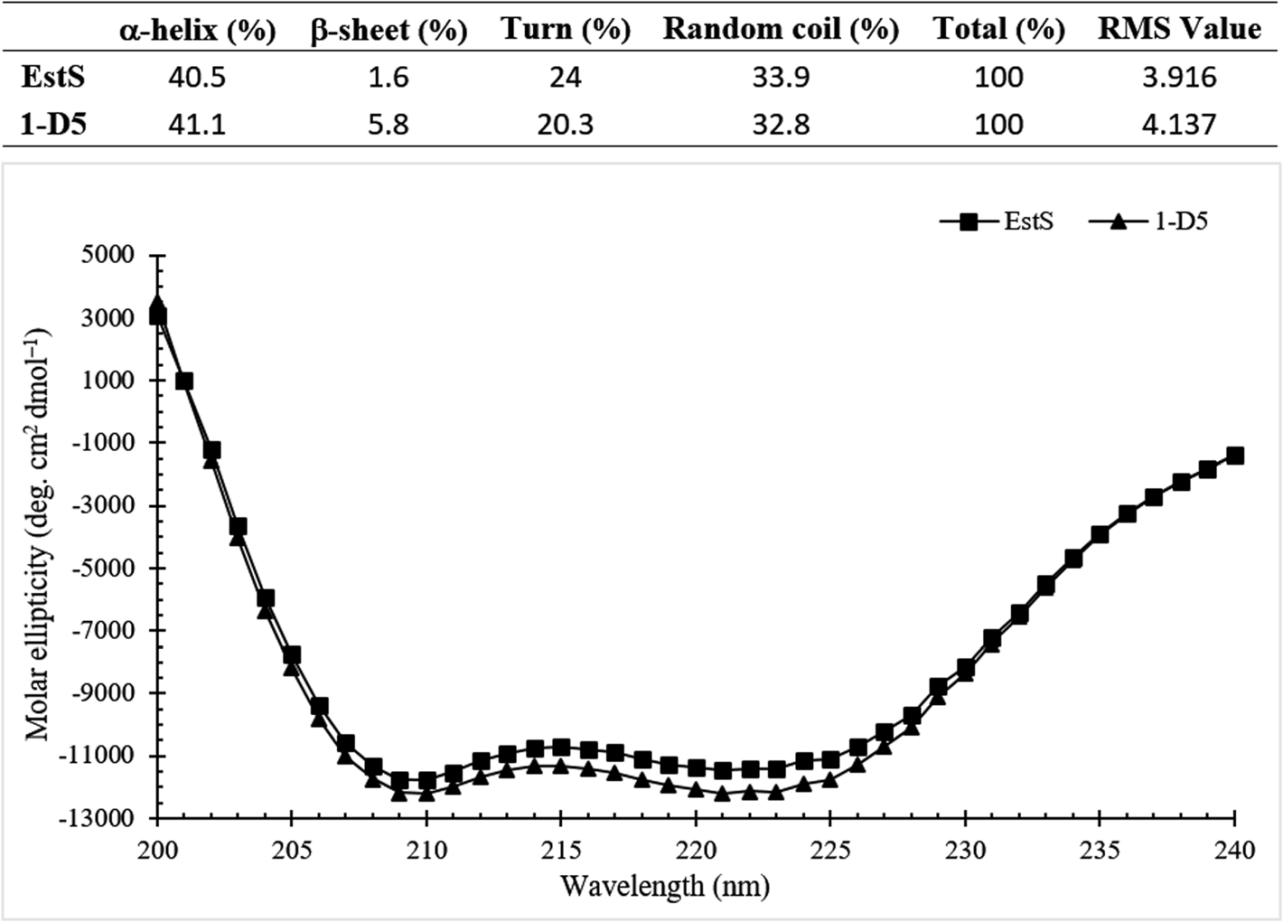
CD spectra of the EstS and 1-D5 in the far-UV spectral region (195–250 nm). CD spectra of Est WT and 1-D5 was more or less similar and an increase in the percentage of β-sheets was reported by CD analysis

The program PSIPRED predicted that the residue Arg116 residue was located in a conservative region. A quantitative analysis of the protein secondary structure for WT and mutant was carried out using SELCON3 program. The data showed that the CD spectra of Est WT and 1-D5 was more or less similar and an increase in the percentage of β-sheets was reported by CD analysis (Fig. 8).

## Discussion

In this study, we have identified and characterized an esterase (EstS) from a marine bacterium *Serratia* sp. EstS preferred short-chain *p-*nitrophenyl esters as substrate and unable to hydrolyze long-chain *p-*nitrophenyl esters (C12 and C16). The specificity towards short chain acyl esters indicated that purified protein (EstS) was an esterase. EstS demonstrated the T_1/2_ of approximately 50 min at 50 °C and retained 41.23 % activity after 1 h incubation. In addition, the activity of EstS WT was increased by low concentration (1 mM) of Mg^2+^ and Mn^2+^, partly inhibited by Cu^2+^ and Zn^2+^, and completely inhibited by the addition of acetonitrile, *n*-butanol and SDS. EstS retain its activity and stability between pH 5.5 and pH 9.5 after 24 h at 4 °C. Furthermore, we also report the engineering of EstS by directed evolution. A thermo-stable mutant 1-D5 was selected from the random mutant library constructed by error-prone PCR. 1-D5 showed change in three amino acids (A43V, R116W, D147N). No change in optimum temperature and pH of 1-D5 was observed in comparison to EstS. But the T_1/2_ at 50 °C of 1-D5 was about 70 min and it retained 63.3 % of activity at 50 °C for 1 h, which were about 22 % higher than WT.

Interestingly, EstS displayed a significant adaptation towards low temperature showing the optimal activity at 10 °C and retained 92 % residual activity at 0 °C (Fig. 4a). However, EstS was considerably unstable at temperatures above 55 °C. These unique characteristics indicate that EstS is a cold-active enzyme (low optimal temperature, poor thermo-stability). The value of optimal temperature is certainly lower than other reported cold-active esterases⁄lipases, such as: EstB from *Alcanivorax dieselolei* B-5(T) which showed optimal activity at 20 °C and retained 95 % of its original activity at 0 °C [19]; Est10 from *P. pacificensis* displayed optimal activity at 25 °C and retained 55 % of its original activity at 0 °C [20]; rEst97 from deep-sea sediment had shown optimal activity at 35 °C and about 12 % relative activity at 0.5 °C [21]; lipase hiLip1 from uncultured microorganism with the optimal activity at 35 °C and 44 % activity at 10 °C [22]. However, the EstS is slightly more stable at high temperature(s) than all these cold-active lipolytic enzymes, which were rapidly inactivated at 55 °C.

The adaptation of cold-active enzyme to low temperatures can be attributed to the conformational flexibility conferred by some structural features: more Gly residues (especially around the active site); less Pro and Arg residues; more Ser and Met [22–24]. Comparatively, EstS has higher percentage of small amino acids Ala (16.56 %) and Gly (8.94 %) than its thermophilic counterpart Est2 (Ala 11.94 %, Gly 7.10 %) from *Alicyclobacillus acidocaldarius* and PestE (Ala 11.94 %, Gly 7.35 %) from *Pyrobaculum calidifontis* [25, 26]. Besides, EstS has less Pro (6.95 %) than Est2 and more Met (2.98 %) than Est2 (0.65 %) and PestE (1.92 %), all this is a factor probably contributing to its adaptation to low temperatures.

Additionally, another noteworthy property of EstS was its strong tolerance to NaCl. EstS was active from 0 to 4 M NaCl and retained nearly 94 % activity even at a salt concentration of 4 M NaCl (Fig. 5a). Unlike most halophlic enzymes which are inactive or unstable under low salt concentrations, EstS was active even without NaCl [27, 28]. This unique characteristic indicated EstS was halo-tolerant rather than halophilic. Furthermore, the presence of NaCl was unable to improve the activity of EstS as compared with other halo-tolerant and/or halophilic lipolytic enzymes, such as: the esterase EstPc from *Psychrobacter cryohalolentis* K5, which showed 179 % activity at 1.75 M NaCl [29]; and esterase PE10 from *Pelagibacterium halotolerans* B2, which exhibited the maximum activity in the presence of 3 M NaCl [30]. Generally, halophilic proteins have a large number of acidic amino acids on the surface, whose negative charge acts to form protective hydrated ion network that keeps the protein stable in high salt concentrations [31, 32]. In the present study, EstS has a higher percentage of acidic amino acid (Asp + Glu: 12.25 %) than the basic amino acid (Arg + Lys: 8.61 %). EstS showed high negative charges on the surface, which is consistent with the distribution of the electrostatic potential of its model (Fig. 7), which clearly indicated that the halo-tolerance of EstS was depends upon structure and amino acid composition. The overall results from this study, suggests that EstS is a novel cold active and halo-tolerant esterase and it may prove useful for immense biotechnological applications.

The major factors responsible for thermostability of proteins includes ionic interactions, hydrogen bonds, hydrophobic interactions and disulfide bonds [33]. In this work, a mutant 1-D5 was more thermo-stable than WT and showed three amino acid changes (A43V, R116W, D147N; Fig. 6). It was predicted that Ala^43^ of EstS is located in the loop near first β sheet (β1) of enzyme and does not directly with the active center. The Arg^116^ of EstS, which replaced by Trp in 1-D5, is located in the loop between fourth β sheet (β4) and second α helix (α2) and the Asp^147^ of EstS is located in the loop near fifth β sheet (β5) and the catalytic residue Ser^157^ is on the other side of β5. Both position 116 and 147 are on the protein surface. The mutation R116W changed the polar amino acid residue to hydrophobic residues while the mutations A43V and D147N tend to increase the hydrophobicity of EstS [34]. This increase in the hydrophobicity lead to higher thermo-stability of 1-D5 than WT.

CD results demonstrated that the mutation of EstS did not affect the secondary structure of enzyme but improve the activity and stability.

## Conclusion

A novel cold-active and salt-tolerant esterase was purified and characterized from marine bacterium *Serratia* sp. EstS showed remarkable catalytic activity at low temperature, extreme salt tolerance and good pH stability. All the characteristics collectively make it a potential candidate for industrial applications. Furthermore, a more thermo-stable esterase was obtained by error-prone PCR, and the experimental results may provide useful information for further study.

## Methods

### Strains, vectors and reagents

*E. coli* DH5α (TaKaRa, Japan) and BL21 (DE3) (Novagen, USA) were used as cloning and expression hosts, respectively. The vector pGEX-6P-1 (GE Healthcare, USA) was used for gene cloning and protein expression. *Serratia* sp. and *E. coli* were grown in LB medium (tryptone 1 %, yeast extract 0.5 % and NaCl 1 % w/v) at 28 and 37 °C, respectively. *p*-nitrophenyl esters: *p*-NP acetate (C2), *p*-NP butyrate (C4), *p*-NP hexanoate (C6), *p*-NP caprylate (C8), *p*-NP laurate (C12) and *p*-NP palmitate (C16) were all purchased from Sigma (St. Louis, MO, USA).

### Gene cloning

The cloning of *estS* gene of *Serratia* sp. was performed by PCR amplification with *estS*-F and *estS*-R primers. The forward primer *estS*-F 5-CCGGAATTCATGCCGCTTGATCCTCA-3 (with *Eco*RI restriction site underlined) and the reverse primer *estS*-R 5-CCGCTCGAGTCAGCCAACCTTGTCGA-3 (with *Xho*I restriction site underlined) were designed based on the sequence of the esterase gene GenBank: AGQ31273.1] of *Serratia liquefaciens* ATCC 27592. The whole genome of *S. liquefaciens* ATCC 27592 [GenBank: CP006252.1] was sequenced and published in GenBank by Nicholson et al [35]. The genomic DNA of *Serratia* sp. was used as template, PCR was performed with an initial denaturation at 94 °C for 3 min; followed by 30 cycles of denaturation at 94 °C for 0.5 min, annealing at 60 °C for 0.5 min, and extension at 72 °C for 1 min; and a final extension at 72 °C for 10 min. The resulting product was digested by *EcoR*I and *Xho*I, ligated into the expression vector pGEX-6P-1 with the same digestion, and then transformed into competent *E. coli* DH5α cells.

### Sequence analysis

GenScript (Nanjing, China) was used to make sure that the gene was correctly inserted sequenced the recombinant plasmid pGEX-6p-estS. The deduced amino acid sequence of *estS* was analyzed by blastp program and the SignalP 4.1 Server (http://www.cbs.dtu.dk/services/SignalP/) predicted the signal peptide. Moreover, the multiple sequence alignment was carried out using Clustal X 2.0.

### Construction of error-prone library

The random mutant library was constructed by error-prone PCR reaction [36]. The 50 μl reaction mixture contained 50 ng of template plamid pGEX-6p-*estS*, 0.2 μM forward primer *estS*-F and reverse primer *estS*-R, 0.2 mM dATP, 0.2 mM dGTP, 0.4 mM dCTP, 0.4 mM dTTP, 5 mM Mg^2+^, 0.3 mM Mn^2+^ and 2.5 U of Taq polymerase. The PCR reaction was carried out under similar condition as the gene cloning of *estS*. The amplified product was digested by *EcoR*I and *Xho*I and ligated in the pGEX-6p-1 vector. The recombinant plasmids were transformed into *E. coli* DH5α cell. The resultant clones were spread onto LB agar plate and incubated at 37 °C.

### Screening of mutants

The mutants were grown for 12 h on LB agar plate and colonies were picked with sterile toothpicks and grown in 96-deep well plates containing 600 μl LB medium supplimented with ampicillin (100 μg/ml) along with WT clone (cell harboring pGEX-6p-*estS*). Each well was supplemented with 150 μl flesh LB-Amp medium containing 1 mM IPTG and T7 phage. The cells were induced and lysed at 28 °C for 6 h followed by incubation at 50 °C for 40 min. Subsequently, the plates were cooled at 4 °C for 10 min, and 20 μl of lysate was transferred to 96-well plates to assay residual enzyme activity at room temperature. The mutants that showed higher residual enzyme activity were selected for further experiments. Finally, the DNA of mutants was isolated, sequenced and compared with the WT to locate the changed amino acid.

### Expression and purification of WT and mutant

The recombinant plasmid pGEX-6p-esterase (WT and mutant) were transformed into *E. coli* BL21 (DE3) and the cells were grown for 12 h at 37 °C in LB medium containing ampicillin (100 μg/ml). When the optical density of the culture reached 0.6 at OD_600_, the cells were induced by adding isopropyl-β-D-thiogalactopyranoside (IPTG; 0.2 mM). After induction for 16 h at 15 °C, the cells were harvested by centrifugation (8000 rpm) at 4 °C for 10 min, followed by washing, resuspension in PBS buffer (0.8 % NaCl, 0.02 % KCl, 0.142 % Na_2_HPO_4_, 0.027 % KH_2_PO_4_; pH 7.4). The disruption of cells was carried out by French pressure cell. The cell lysate was collected by centrifugation (12,000 rpm) at 4 °C for 40 min. The supernatant was purified by Glutathione-Sepharose column (GE Healthcare, USA) as described previously [37]. Finally, the target protein, wild-type and mutant esterase was released from the GST-tag attached to the column by the 3C protease solution (10 U/μl, PreScission, Pharmacia). The purified protein was analyzed by SDS-PAGE in 12 % polyacrylamide gels. Protein concentration was determined by the Bradford method [38], using bovine serum albumin as standard.

### Enzyme assay

The esterase activity was determined by measuring the amount of *p*-nitrophenol released in the standard reaction mixture with the OD values at 405 nm monitored by Thermo Scientific Multiscan Spectrum. The standard reaction mixture contained 10 μl of enzyme, 2 μl of *p*-NP ester (20 mM) in ethanol and 188 μl of Tris–HCl buffer (50 mM, pH 8.5). The reaction mixture in which enzyme is replaced with PBS was considered as control. All experiments were carried out in triplicate and the data obtained were analyzed using GraphPad Prism 5.0 and Excel 2010 software. The calculated values were expressed as mean ± standard deviation (SD) with the statistical significance at *p* < 0.05 and standard deviation was calculated by standard deviation function (STDEV) in Excel. One unit of enzyme activity was defined as the amount of enzyme needed to release l μmol of *p*-nitrophenol per minute under the above reaction conditions. The experiments were performed in triplicate and average values were calculated with standard deviations.

### Substrate specificity

The substrate specificity was investigated with *p*-NP esters of different chain lengths (acetate, C2; butyrate, C4; hexanoate, C6; caprylate, C8; laurate, C12; palmitate, C16). The hydrolytic reactions were performed in triplicate under standard assay conditions with each substrate.

### Biochemical characterization of esterase

The optimum temperature of esterase was determined by incubating the reaction mixtures at a temperature range of 0 to 80 °C with *p*-NP acetate (C2) as substrate. The thermal stability was determined by measuring the residual enzyme activity after exposing the enzyme solution separately to three different temperatures (45, 50 and 55 °C) for varying time intervals. The pH optimum was investigated in following buffers: phosphate–citrate buffer (pH 5.0–7.0) and Tris–HCl buffer (pH 7.0–10.0). The pH stability was evaluated by incubating the enzyme in various pH buffer solutions for 24 h at 4 °C. The remaining enzyme activity was assayed under the standard conditions.

The effects of metal ions (Mg^2+^_,_ Sr^2+^, Ba^2+^, Zn^2+^, Mn^2+^, Cu^2+^, Ca^2+^) and reagents (EDTA, PMSF) on esterase activity were examined at a final concentration of 1 and 5 mM in Tris–HCl buffer (pH 8.5). The effects of organic solvents and detergents were evaluated by diluting the enzyme solution in different final concentrations (10–30 %) of organic solvents and detergents, including *iso*-propanol, acetone, methanol, DMSO, ethanol, *n*-butyl alcohol, ethylene glycol, acetonitrile, TritonX-100, Tween-20, Tween-80, CHAPS and SDS. Samples containing only the same amount of reagent were used as control. The enzyme activity without additives in the reaction mixture was considered as 100 %. The effect of NaCl on enzyme activity was determined with 0–4 M NaCl dissolved in Tris–HCl buffer (50 mM, pH 8.5). The effect of NaCl on enzyme stability was evaluated by treating enzyme solutions in the above-mentioned NaCl solutions at 4 °C for 24 h.

### Kinetic measurements

The kinetic parameters (*k*_*cat*_, *V*_max_, and *k*_m_) were determined by measuring the reaction rate of WT and mutant in different substrate (*p*-NP acetate) concentration (0.01–0.3 mM) at 10 °C for 10 min. The kinetic parameters *V*_max_ and *k*_m_ were determined by Lineweaver-Burk plot using Michaelis–Menten equation with Graphpad Prism software (Graphpad, San Diego, CA). The *k*_*cat*_ parameter was calculated using the equation *k*_*cat*_ = *V*_max_/[E].

### Homology modeling

To gain insights into the structure of WT and mutant esterase, a homology model was generated automatically with Swiss-model server (http://swissmodel.expasy.org/; [39]. Based on the model, the electrostatic potential on the surface of esterase was visualized by the software SYBYL and the amino acid changes were analyzed by MOE 2009.

### Circular dichroism analysis

Secondary structure of Est WT and 1-D5 was predicted by using the program PSIPRED [40]. Circular dichroism (CD) spectra of Est WT and 1-D5 were recorded with a Jasco-810 CD spectrometer (Jasco Corp., Japan). The data were collected at room temperature from 195 to 250 nm using 2 mm quartz cuvette (600 μl). The conversion to the Mol CD (∆ε) in each spectrum was performed with the Jasco Standard Analysis software. Estimation of the secondary structure content from far-UV circular dichroism (CD) spectra was performed by using the CDPro software package (available at http://lamar.colostate.edu/~sreeram/CDPro/main.html), including three executable programs (SELCON3, CDSSTR, and CONTIN/LL) [41]. In this study, the percentages of α-helix and β-sheet for each protein sample were averaged by the calculations of results from the CDPro software package. The circular dichroism data were expressed in terms of the mean residue ellipticity (θ_mrw_),which calculated with using the equation [42]:

$$ {\uptheta}_{\mathrm{mrw}}=\frac{M_w\cdot {\uptheta}_{\mathrm{obs}}\cdot 100}{\mathrm{N}\cdot d\cdot c} $$

where θ_obs_ is the observed ellipticity in degrees, Mw is the protein molecular weight of WT and 1-D5, and N is the number of residues, *d* is the path length of quartz cuvette (0.2 cm), *c* is the protein concentration (mg/ml), and the constant number 100 stems from the conversion of the molecular weight to mg/dmol.

### Availability of supporting data

The additional data supporting the results of this article is available online in the National Center for Biotechnology Information [NCBI] repository, [GenBank accession number:KU362566].
